# Driving for patients in palliative care–a reality?

**DOI:** 10.1186/2193-1801-3-79

**Published:** 2014-02-10

**Authors:** Anders Widman, Stefan Bergström

**Affiliations:** Palliative Care Team, Department of Oncology, Gävle Hospital, Gävle, Sweden

## Abstract

**Objective:**

Very little is known about car driving in palliative patients. We have asked patients in at home palliative care environments about their driving habits during palliative care.

**Methods:**

At admittance to the palliative care program and at regular visits the patients were asked a few questions about age, diagnosis, sex, driving license, driving, medication and functional status.

**Results:**

23% (40) of our studied patients (173) were still driving, and if we included patients who responded that they would still consider driving, the corresponding figure was 39%.

**Conclusions:**

Our study indicates that the prescribing doctor should be well informed about national driving legislation and be prepared to discuss driving in combination with heavy opioid medication and tranquilizers even with patients in palliative care.

## Introduction

Quality of life is important for patients who are suffering from life-threatening diseases and their families. In palliative care, symptom relief, teamwork, communication and attention to relatives/carer are important aspects of helping patients maintain their dignity and well-being. Palliative care is often introduced in end-of-life care, in order to attend to these patients’ physical, psychological, social and existential needs. Although studies are increasingly being done on this patient group, there are still concerns that have not been addressed.

We know that car driving is important to the lives of most people in Sweden, but we do not know how common driving is among our palliative patients. How does their palliative status affect their driving? How many patients in palliative care still drive? What medications do they take? Is there a sex role difference? How important is it to these patients to retain their drivers’ licenses? Being able to drive is one aspect of maintaining integrity, giving the patients some freedom despite a life-threatening disease. Having a car is also often part of the patient’s being able to stay at home despite the disease.

A literature search for studies on palliative care and driving turned up no hits.

To our knowledge this is the first study on this topic. The present paper describes car driving habits during end-of-life care.

## Materials and methods

The palliative care team in the county of Gävleborg serves patients in urban and rural areas. The county is just over 4,000 km^2^ and is located in central Sweden. The palliative team sees patients on heavy medication, such as opioids and tranquilizers.

Our routine is to accept patients primarily with cancer diagnoses and living in their own homes, sometimes supported by family, friends, home care and local nurses. We see the patients at home by agreement, usually once every three or four weeks, and by need, and we are in close contact by telephone. The patients are mainly referred to palliative care from departments of oncology, internal medicine (mainly with lung cancer), surgery and gynecology. The patients are almost always handed over to the palliative care team by their referring doctor, including GP:s, after referral.

During one year we have approximately 200 deaths among our approximately 300 referred patients per year, and a mean survival time of 3–4 months, with large variations. The difference consists of patients actually not coming to their homes again, dying before enrollment and a slow increase in patient survival time.

For this study we asked 200 consecutive patients, both new patients at the introductory appointment and existing patients at follow-up appointments to answer a questionnaire about their driving habits. Those who responded that they were still driving were asked to fill out the questionnaire again at the next appointment. The study began on September 15, 2011 and the last patient was included on March 1, 2012. The study was completed at the end of April 2012. The study was approved by the Ethics Committee at Uppsala University (Dnr 2011/098).

The inquiry consisted of questions about age, diagnosis, sex, driving license, driving, medication and performance status. The questionnaire was discussed and filled in by patient, often with comments from relatives at our regular visits.

## Results

Of the 200 patients initially asked, 27 were not included in the study for various reasons such as poor mastery of Swedish, dementia, too poor performance status to be asked again or imminently death. Only one person did not agree to participate in the study.

The most common diagnoses are listed in Table 1. Note that in the lung cancer and breast cancer groups there were no patients who were still driving.

**Table 1:** **Diagnoses of the patients in the study**

Diagnosis	Total (n)	Drivers
Prostate cancer	25	12
Lung cancer	23	0
Colon cancer	23	7
Rectal cancer	12	6
Pancreatic cancer	12	3
Breast cancer	11	0

A total of 173 patients were included, 90 men and 83 women (Table 2).

**Table 2:** **Age and sex of patients in study**

	Men	Women
Number (n)	90	83
Mean	75 y	74 y
Median	77 (46–91 y)	76 (41–94 y)
No drivers’ license (n)	5 (6%)	25 (31%)

Forty (23%) patients, of whom seven were women, initially said that they were still driving. The mean age was 65.6 years for the women and 71 years for the men. Another group of 27 patients (10 women) still considered driving an option although they did not exercise it (Table 3).

**Table 3:** **Patients still driving and patients considering driving still an option**

	Men	Women
Drivers (n)	33	7
Mean age	71 y	65.6 y
Consider driving an option (n)	17	10

These groups of actual and potential drivers accounted for 39% of all included patients. We later found out that 9 patients (4 women) had said they were still driving were actually not, according to later information from their relatives, friends, home care and local nurses.

Of the 52 men who said they were not driving, 27 had driven during 2011 prior to study entrance. The corresponding figure for women was 15 out of 50.

Almost all the patients with a drivers’ license considered it important to keep their licenses for reasons ranging from identification, to hope, to emergencies. The patients in our study who had drivers’ licenses had had them for a long time: 24–73 years (median 50 years).

Performance status (WHO) was significantly better among drivers than non-drivers (Table 4).

**Table 4:** **Performance status (WHO) at inclusion**

Average	Man	Woman
Driver	1.6	1
Non-driver	2.2	2.2
**Performance status (WHO) for our patients outside the study two different years at our unit**		
Average at enrollment	2007	2011
	2.6	3.0

Of the patients who did not drive during the study, 43% died during this study period. The corresponding figure for participants who were still driving was 48%.

With regard to driving frequency, 13 patients responded that they were still driving daily and 20 others that they were still driving 1–4 times per week. A few who were still driving drove less frequently. There were no differences between those living in urban and rural areas. Of the 40 patients still driving, 17 said they had no one to help them with their driving needs, and no participants used the transportation services for the disabled.

In the group who were still driving 26(65%) were on opioids in form of fentanyl-patch, oxycodone and long-acting morphine. Doses varied from 24 to 250 ug of fentanyl (oral morphine 60–860 mg), oxycodone 10 to 80 mg (oral morphine 20–160 mg) and long-acting morphine 20 to 120 mg (Table 5).

**Table 5:** **Medication among drivers**

Medication	Number (n)
Fentanyl patch	8
Long-acting morphine	9
Oxycodone	5
Morphine	11
Morphine if needed	5
Antidepressants	33%
Hypnotics	20%

Antidepressants were in use among 33% of driving patients. Eight (20%) were on medication for insomnia and 13 (33%) were on sedatives either regularly or when needed (Table 5). Seven of the driving patients were on no medication at all.

## Discussion

Swedish driving legislation says that it is not allowed to drive using medicine classified as narcotics if it is not prescribed by doctors and if you can not handle ordinary traffic situations safe. That is also true for other medication.

In this investigation, we found no previous studies concerning car driving and palliative care patients. We found that none of our patients with a diagnosis of lung cancer or breast cancer were still driving. Patients with these diagnoses had higher performance status (WHO) than average, probably owing to late remittance and long cancer treatment.

Non-inclusion was as expected, owing to cultural problems, dementia, and patients too ill to be included, i.e. with death imminent, which correlates to our previous knowledge of this patient cohort, where 25% of the palliative care patients die within one month. Since our follow-up was only at three to four week intervals, this made it impossible to follow the development of driving habits in this patient group.

Most of the men and women in our study had a drivers’ license and intended to keep it, considering it related to their integrity. The women in our driving group seemed to be more cautious and had better performance status. Having reasonable performance status does, however, not preclude rapid changes, and 48% of the drivers died during the study. This is an indication that there is often a short time lag between driving and non-driving.

We also have indications that patients’ performance status and even their ability to drive may improve after being included in palliative care. This may be due to stopping cytostatic treatment or other interventions which are known to be fatiguing. Performance status was better, measured when included in the study than at inclusion in routine palliative care outside the study (Table 4).

Medication status was very similar between drivers and non-drivers, and between men and women. Studies concerning effects on driving ability while on strong pain killers, antidepressants and sedatives are sparse. There are very few such concerning cancer patients and none concerning palliative care patients. There are indications that stable medication with opioids and antidepressants does not affect driving ability (Vainio et al. 1995; Wingen et al. 2005). Such studies mainly seem to have been made on subjects without life-threatening diseases. Most patients who are prescribed sedatives to be taken when needed often seem not to take them at all while still driving, but there are to our knowledge, no such studies concerning patients on palliative care, (Vainio et al. 1995; Wingen et al. 2005; Johansson 2011/2012).

Our driving patients lived both in urban and rural areas and half of the drivers said they would have had no one else to help them with driving if they stopped.

One group of patients with drivers’ licenses had made a definite personal decision not to go on driving, and they were often able to state exactly when they had made this decision, and why. Poor general health status was often mentioned. We could also see that one group of our patients who considered themselves drivers were actually in the process of losing their autonomy, although they had not yet acknowledged it.

No accidents came to our attention, but one male patient was reported to the authorities for not agreeing to stop driving although he had been instructed by the doctor to stop. He turned out to have brain metastases.

### Clinical implications

We found driving to remain important, even for patients in palliative care, a fact which is useful to know in clinical practice. Continuing to drive is one aspect of maintaining hope, a feeling shared by patients in palliative care. The individual’s performance status is important in relation to his or her ability to drive safely and can change rapidly. Our team tries to recommend our patients to avoid driving feeling fatigue. Doctors who see these patients should be familiar with driving legislation in their country in order to be able to advise their palliative patients in the best manner possible. They should also learn about conclusions drawn from research in this field and find compilations for local conditions, as in Sweden (Johansson 2011/2012). If available they could use written patient information (Patient information 2009).
